# Direct recycling of end-of-life lithium-ion batteries cathode active materials by hydrothermal route

**DOI:** 10.1038/s41598-026-41973-7

**Published:** 2026-03-02

**Authors:** Juan Castro, Marta Gómez, Pedro J. Acebes, Paula Moretti, M. R. Bermejo, Maximiliano Merlo, Yonca Belce, Jordi J. Biendicho, Silvia Bolado-Rodriguez, Dolores Hidalgo

**Affiliations:** 1 CARTIF Technology Centre, Area of Circular Economy, Boecillo, Valladolid, 47151 Spain; 2 Institute of Sustainable Processes, University of Valladolid, Valladolid, 47011 Spain; 3 Catalonia Institute for Energy Research (IREC), Sant Adrià de Besòs, Barcelona, 08930 Spain

**Keywords:** Annealing, Battery Recycling, Cathode regeneration, Circular Economy, NMC622, Re-lithiation, Chemistry, Energy science and technology, Materials science

## Abstract

At the present time, the increasing use of lithium-ion batteries in electric vehicles has created unprecedented pressure for end-of-life management and resource recovery. This article reports on a direct recycling approach to regenerate spent cathode active materials, in particular Ni-rich NMC622, via a hydrothermal re-lithiation strategy and thermal annealing. An initial screening process was established to separate high purity spent cathode active materials from disassembled LG Chem pouch cells from Hyundai KONA battery packs. A full factorial design was applied to provide a meaningful statistical analysis of the influence of hydrothermal variables - LiOH concentration, temperature and reaction time. The results indicate that lithium concentration and temperature have a strong main effect on regeneration efficiency, while interaction effects with time are more influential for lithium incorporation. The regenerated cathode active materials exhibited structural, morphological and electrochemical performance comparable to commercial NMC622, especially for samples treated at 160 °C, 4 M LiOH and 1 h reaction time. This process demonstrates the feasibility of regenerating degraded cathode active materials for reuse in new batteries, contributing to circular economy strategies and critical raw material independence in Europe. On the other hand, detailed material characterization validated the recovery of layered crystalline structure and localized cation mixing, conditions required for best battery performance. Regenerated electrodes retained high specific capacity during electrochemical testing and displayed good stability over 50 cycles under the conditions tested. Interactions were quantitatively significant and through the statistical analysis approach, optimal synthesis conditions were directed based on interaction limits. Against this background, the proposed method circumvents the high energy consumption and material losses of the pyrometallurgical route and the secondary pollution and reagents needed in the hydrometallurgical leaching process. In summary, direct recycling appears to be a more resource-efficient and sustainable route for the recovery of cathode materials in future battery supply chains.

**Supplementary Information:**

The online version contains supplementary material available at 10.1038/s41598-026-41973-7.

## Introduction

In the search for new renewable energy solutions, Lithium-Ion Batteries (LIBs) are one of the most promising technologies for energy storage, offering high energy density, long life, and multidisciplinary applications. Thanks to these advantages, LIBs have been perfectly integrated into stationary use in homes and industry and for powering the electric motors of heavy vehicles or light personal transport. The scientific community’s intense research into the vagaries of lithium-ion chemistry has catalysed remarkable innovation and presented numerous paradigms to elucidate the fundamentals of LIB technologies in their ever-evolving permutations^1^^2^,.

Inside LIBs, there is an interplay of cathode and anode materials, electrolytes and separators. This ensemble allows lithium ions to move effectively and reversibly between the anode and cathode during charging and discharging^3^. However, new electrode materials, electrolyte formulations and manufacturing processes have been required to improve performance and sustainability for the next generation of LIBs. Researchers face a myriad of issues ranging from capacity degradation to safety and the environmental footprint of raw materials which require integrating sustainability across the entire battery life^4^^5^, . From advanced recycling technologies that recover valuable materials from spent batteries to the search for environmentally friendly alternatives in manufacturing, these solutions underscore the scientific community’s commitment to creating a sustainable and circular LIBs value chain, where advances in design, materials and recycling technology converge to deliver minimal environmental impact and maximum resource efficiency.

**Fig. 1:**
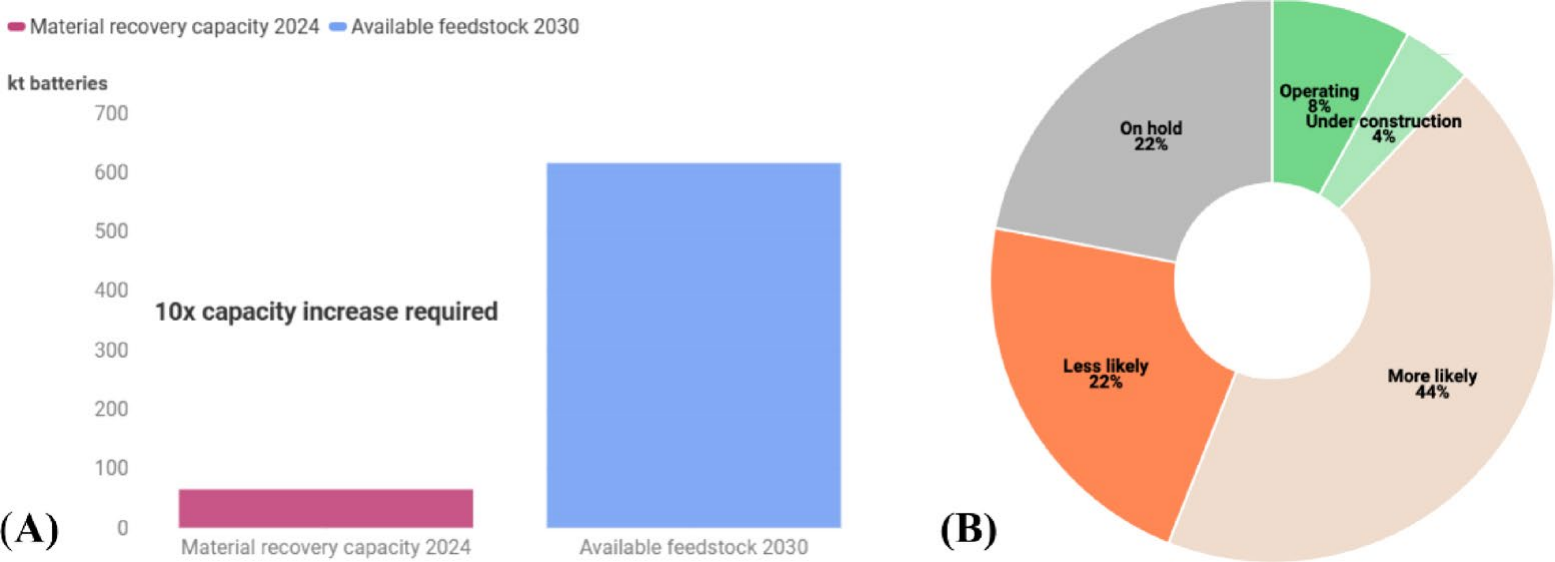
**(A)** Current recycling capacities and future feedstock by 2030. **(B)** Status of announced material recovery projects in Europe. Source: Adapted from^6^.

Battery recycling offers Europe a significant opportunity to secure critical raw materials, reduce environmental impacts, and advance resource independence in the electric vehicle (EV) sector. By 2030, recycled batteries and production scrap could supply up to 25% of cobalt, 17% of manganese, 16% of nickel, and 14% of lithium, enabling the production of 1.3–2.4 million EVs and avoiding the need for 12 new mines globally by 2040^6^. Recycling also cuts CO₂ emissions, with recycled lithium producing 19% fewer emissions than conventional extraction. However, Europe’s current recycling capacity is 10 times lower than needed (Fig. 1A), with nearly 44% of planned projects at risk due to high energy costs and financial barriers (Fig. 1B). Scaling up infrastructure, advancing technologies, and enforcing policies like the EU Battery Regulation and Critical Raw Materials Act are essential to unlocking the full potential of a circular battery economy^6^.

As mentioned, the end-of-life (EoL) management of LIBs is a critical mainstream consideration as a major secondary source of valuable materials such as cobalt, nickel, manganese, lithium and graphite, among others. Their recycling is currently a non-sustainable process, solved by non-100% green technologies such as pyrometallurgy^7^ or inorganic acid leaching^8^, which do not reduce the battery footprint and produce secondary wastewater, respectively^9^. However, good recovery rates per critical raw material can be achieved, depending on the type of LIB. Recently, there has been a move away from the conventional and destructive acid-leaching process towards direct recycling. Direct recycling, a method of regenerating degraded cathode material without compromising its original structure, is gaining interest among the battery community. This approach represents a significant advancement, with various techniques such as electrochemical re-lithiation^10^^,^^11^, solid-state synthesis^12^^,^^13^, and hydrothermal re-lithiation^14^^,^^15^ gaining prominence for their enhanced sustainability and reduced environmental impact. Thus, direct recycling is becoming more attractive to researchers and recycling stakeholders due to its high recovery rate and improved sustainability, but large-scale developments are not yet underway.

The current cathode active material (CAM) market is dominated by NMC materials. These materials are layered oxides with a NaFeO_2_-type structure characterised by alternating layers of transition metals (Ni, Mn, and Co) and Li ions from which Li^+^ can move in and out of the structure over many cycles resulting in high battery efficiency and capacity. Intercalation materials such as NMCs have an open framework that facilitates the movement of lithium ions. They also exhibit good electronic and ionic conductivity, enabling efficient migration of Li^+^ and electrons during operation. In addition, they undergo minimal structural changes during intercalation and deintercalation, which contributes to the long lifespan for Lithium-ion batteries. From the original work conducted by Prof. John B. Goodenough^16^, who discovered LiCoO_2_ as a Li-ion intercalation material in 1980, many materials have been proposed and implemented as cathode^17^, including NMC622, which stands for the chemical formula LiNi_0.6_Mn_0.2_Co_0.2_O_2_. This is a very popular cathode material that contains a significant amount of nickel, so discharge capacity is enhanced, but this affects the crystal structure and defect content of the material^18^, including, for instance, Li and Ni site exchange in the crystal lattice^19^. Considering that global battery demand is expected to grow by 25% per year to reach 2,600 GWh in 2030^20^, the need for efficient industrial recycling solutions is critical. With this in mind, research projects worldwide are exploring cutting-edge technologies to enhance recycling rates and diminish the environmental impact of the battery industry. This study proposes a feasible strategy of hydrothermal re-lithiation on spent NMC cathode materials to perform regenerated CAM. The role of the pre-processing step in this proposed direct recycling process is a key point because it provides pure spent CAM free from anode components, metal current collectors, binders, neither electrolyte salts. This increases the electrochemical performance of the regenerated CAM for its reuse in a new battery with recycled components. Therefore, this economical and environmentally friendly method is expected to be used for large-scale recycling of spent CAM materials in a near future, as it would be applicable to different types of cathode chemistries.

To better position the contribution of this work with respect to the state of the art, it is important to emphasize three aspects. First, the study starts from real end-of-life EV cells (LG Chem pouch cells from Hyundai KONA packs) and includes a practical pre-processing step to obtain high-purity spent cathode active material. Second, a full factorial experimental design is applied to quantify the main and interaction effects of LiOH concentration, temperature and reaction time on lithium reincorporation. Third, the regenerated powders are directly benchmarked against a commercial NMC622 reference using structural and electrochemical characterization, enabling a clear process-structure-performance discussion.

Hydrothermal re-lithiation is a promising method for direct recycling spent LIBs. This process involves treating spent cathode materials with a solution containing an excess lithium source, allowing the added lithium to integrate into the structures of the spent cathodes^8^. It is a highly controlled process, and key parameters, such as temperature, pressure and reaction time, must be precisely controlled and adjusted. Those conditions are crucial for achieving the successful re-lithiation of CAM.

**Fig. 2:**
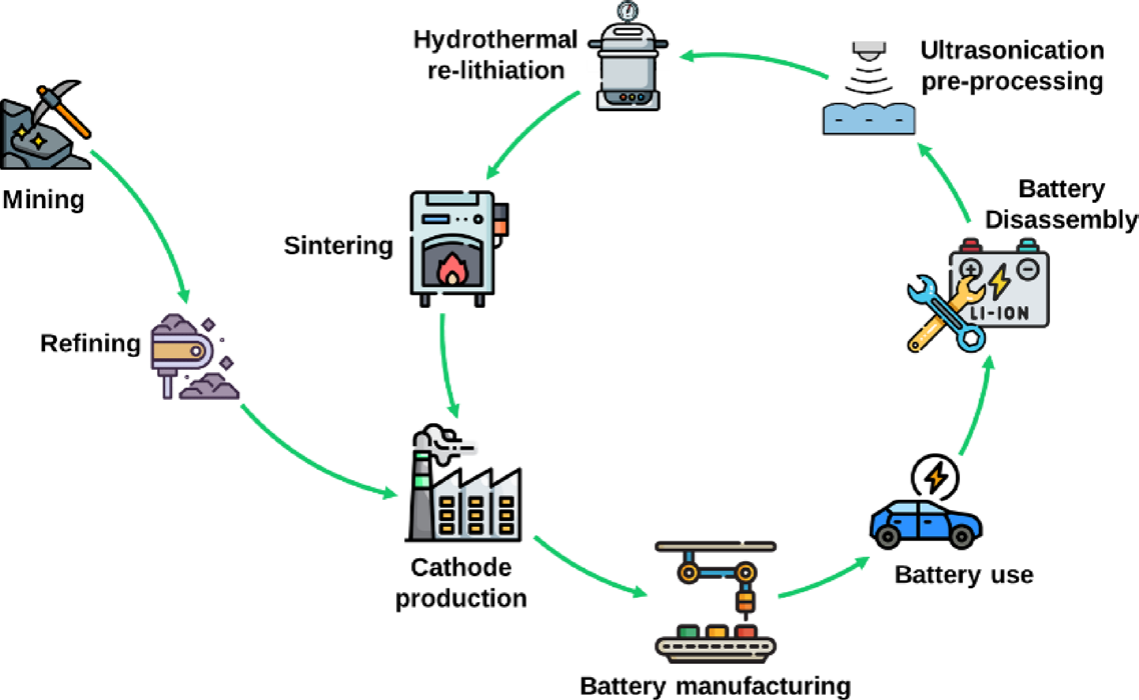
Integration of direct recycling through hydrothermal route inside battery value chain.

The re-lithiation reaction occurs when lithium-deficient cathode material (Li_x_Ni_y_Mn_w_Co_z_O_2_) incorporates lithium ions (Li^+^) from the lithium source solution, LiOH in this case. This process leads to the formation of a fully lithiated cathode material (LiNiyMnwCozO_2_), and the reduction of the OH- ions produces water and oxygen gas (Fig. 2). The Eqs. 1 and 2 shows the redox reactions during the re-lithiation of the Li_x_Ni_y_Mn_w_Co_z_O_2_ material. The proposed mechanism for the hydrothermal re-lithiation reaction is illustrated in Figure S1.

1 $${\rm 4OH^-_{(ag)} \rightarrow O_{2(g)} + 2H_2O_{(1)} + 4e^-}$$

2 $${\rm Li_x Ni_y Mn_w Co_zO_{2(s)} \:+\: (1-x) Li^+_{(aq)}\: +\: (1-x)e^- \rightarrow \: LiNi_yMn_wCo_zO_{2(s)}}$$

This approach enhances the efficiency and sustainability of battery recycling, supporting the circular economy by reducing the demand for new raw materials and minimizing waste.

## Materials and methods

### Chemicals and materials

Lithium hydroxide anhydride (99.7%, Alfa Aesar), hydrochloric acid (34–37%, Fisher Chemical) and nitric acid (67–69%, Fisher Chemical) were used during the experiments. Degraded NMC batteries were obtained from a Hyundai KONA battery pack.

### Cathode material pre-treatment

To ensure safety, the electric vehicle Hyundai KONA battery pack was first discharged before start disassembling operation. The spent battery cells (LG Chem E63B) were manually disassembled into cathode electrode foil, anode electrode foil and separator (Figure S2). Subsequently, the foils were dried in a vacuum drying chamber (Binder VDL 56) to condensate the electrolyte. To obtain the powder spent cathode material, the aluminium collector was delaminated using ultrasonic treatment (Weber Ultrasonics Generator LC Premium). The spent cathode material was then filtered and dried at 90 °C for 24 h. Once the cathode material was dried, it was used for the following direct regeneration experiment.

### Material regeneration: Hydrothermal process and annealing

To restore the spent cathode materials to their original structure and composition, a two-step treatment, hydrothermal re-lithiation and short annealing, was applied to effectively recover their electrochemical properties. For this process, 50 g/L of spent cathode material was added to a reactor (Parr 4520) containing lithium hydroxide (LiOH) solution with concentrations ranging from 0.5 to 4.0 M. The reactor was heated to a temperature range of 160–220 °C for 1 to 2 h, with continuous stirring at 300 rpm. Once the reaction was completed, the product was filtered, washed with deionized water to remove unreacted lithium and impurities, and then dried at 90 °C for 24 h. The annealing process was performed in a muffle furnace for 4 h at 850 °C. The product obtained after these steps was the regenerated cathode material, which was used to fabricate new LIBs to evaluate the electrochemical performance.

### Material characterization

The chemical composition of the solid samples was determined by leaching in concentrated HCl/HNO_3_ solution mix at 210 °C for 1 h in a microwave digestion system (MARS 6). The concentrations of Li, Ni, Mn and Co from the leachate solution were determined using inductively coupled plasma mass spectrometry (ICP-MS Agilent 785). The surface morphology of the samples was analysed using scanning electron microscopy (SEM, Thermofisher Prima E) equipped with an energy-dispersive X-ray spectroscopy (EDS) detector. The crystal structure of the spent CAM, regenerated cathode material and commercial NMC was characterized using X-ray diffraction using a Bruker D8 Advance diffractometer with Cu-Kα (λ = 0.15406 nm).

### Electrochemical characterization

The NMC electrodes consist of 90 wt% regenerated active cathode material, 5 wt% super P carbon (Thermo Fisher Scientific, > 99%) and 5 wt% polyvinylidene fluoride (PVDF, MTI, > 99.5%) binder dissolved in N-Methylpyrrolidone (NMP, Sigma-Aldrich, > 99%). The slurry was mixed in a ball mill at 20 Hz for 15 min and coated on an Al current collector using the doctor blade technique to achieve homogeneous and crack-free electrodes from the recycled and commercial NMC622 (MTI) materials. The electrodes were stored in a vacuum oven at 110 °C for 24 h prior to cell assembly. Cathode discs of 12 mm were used to assemble half-cells (vs. Li metal) coin-cells with dimensions 2032. The mass loading of the regenerated NMC electrodes was 3.60, 3.86 and 3.08 mg/cm^2^ for M1, M2 and M8 respectively. The loading of the commercial electrode was 3.5 mg/cm^2^.

Coin cells were assembled in a glovebox under an inert atmosphere with oxygen and water levels lower than 0.01 mg/L. The prepared regenerated electrodes, lithium metal and a microporous 2400 membrane (Celgard) were used as the cathode, the anode and the separator, respectively. The electrolyte was 1 M LiPF_6_ in ethylene carbonate (EC)/diethyl carbonate (DEC) (50/50 vol%, Sigma-Aldrich, battery grade). All the assembled cells were rested for 8 h before electrochemical testing at room temperature. Galvanostatic charge-discharge was carried out using a multichannel battery test system (Netware). The cells were activated by applying three formation cycles at 0.05 C and cycled at different C-rates (1 C = 160 mAh/g) via constant current mode in the voltage ranges of 3.0–4.3 V versus Li/Li^+^. Electrochemical impedance spectroscopy (EIS) tests were carried out in the frequency range of 10 mHz to 10 kHz with amplitude voltage of 20 mV by EC Lab software (Biologic).

### Statistics analysis

 In order to achieve the optimal conditions of the hydrothermal process, a 3 × 2 × 2 full factorial design without replication was carried out. The data were analysed using a three-way ANOVA to discern both main and interaction effects. Therefore, this analysis evaluates how the three variables (temperature, reaction time and lithium solution concentration) interact to influence in the composition of cathode material. A total of 12 combinations (or tests) were carried out (Table S1) and the cathode material obtained in each test were identified as “M” followed by the test number. The dependent variable was the composition of cathode material, and the independent variables included concentration, temperature, and reaction time, as well as their two-way interactions. Due to the lack of replication, the highest-order interaction (Concentration × Temperature × Reaction time) could not be estimated separately.

## Results

The proposed direct recycling of spent CAM from electric vehicle LIBs is shown in Fig. 2, highlighting its place in the value chain. Unlike other research, this work starts from a real scenario of degraded batteries. As previously mentioned, Hyundai KONA batteries were first fully discharged and then disassembled to separate the electrodes. This was followed by an ultrasonic pre-processing step to separate the spent CAM from the aluminium current collector. The solid fraction was used in the hydrothermal reactor by adding the lithium source for its restoration and further annealing to obtain the restored CAM ready for cathode production and battery manufacturing, thus closing the loop and reducing the mining consumption.

### Spent cathode material characterization

To compare the experimental design results and calculate the efficiency of hydrothermal re-lithiation, the baseline metal content of the initial cathode powder was determined. The chemical composition of the spent CAM was analysed by ICP-MS and the results are presented in Table 1. From the measured Li/(Ni + Mn+Co) molar ratio (0.928 versus the ideal value of 1.000 for a fully lithiated layered oxide), the spent CAM shows an apparent lithium deficiency of ~ 7.2%. The relatively large deviations reported for Li and Co reflect the combined effect of sample heterogeneity and analytical uncertainty (microwave digestion, dilution and ICP-MS quantification), and do not change the conclusion that lithium loss is the dominant compositional deviation in the spent material. Lithium inventory loss during battery ageing is commonly associated with parasitic reactions and SEI growth on the graphite anode, which consume cyclable lithium^21^.

**Table 1:** Composition of the spent CAM.

	Li	Ni	Mn	Co	Li/(Ni + Mn+Co)
Composition (wt %)	6.210 ± 0.966	36.928 ± 0.492	10.600 ± 0.455	8.379 ± 1.199	0.928

**Fig. 3:**
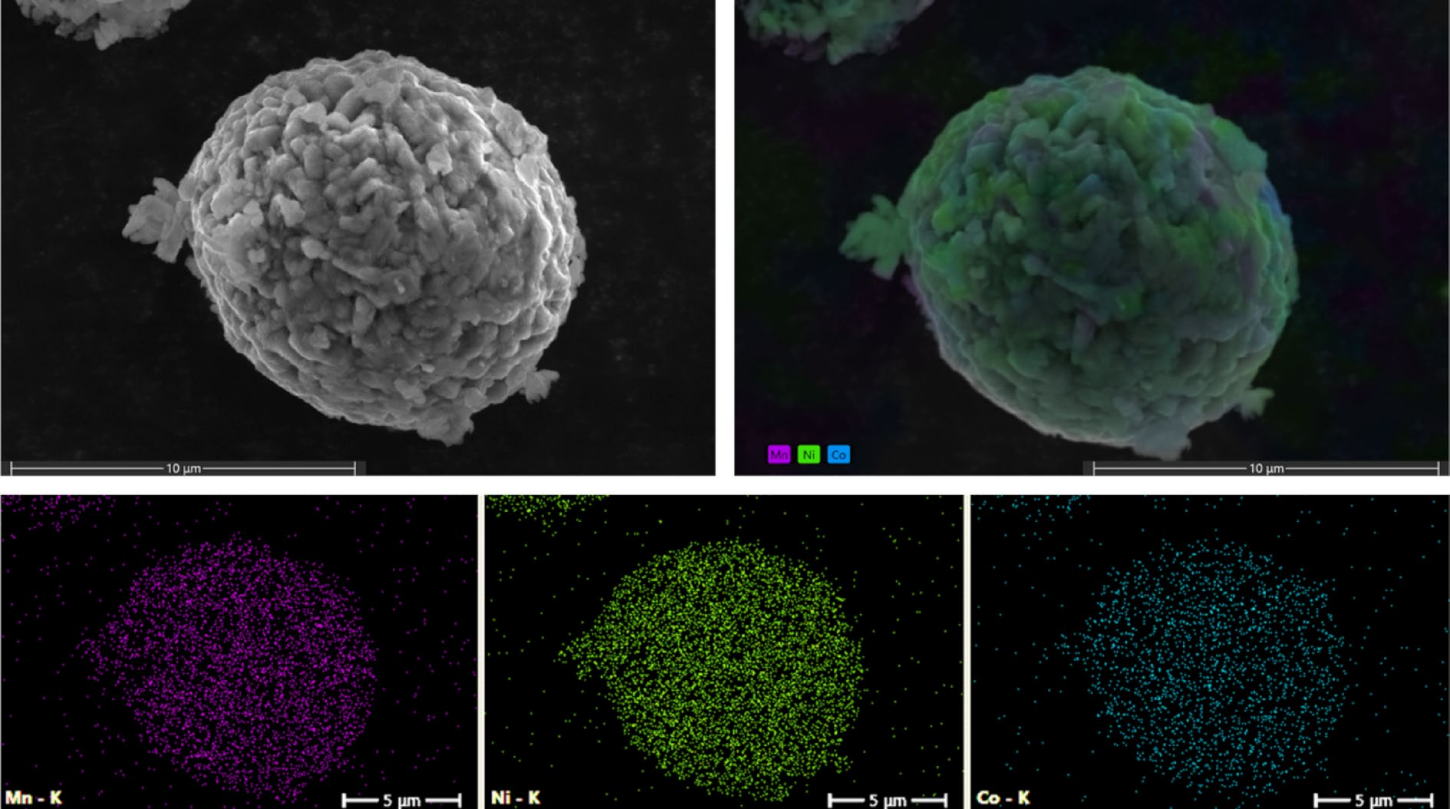
SEM images of the spent CAM and its corresponding SEM-EDS elemental map.

The morphology of the spent CAM was characterised using SEM and EDS elemental map to study its structure (Fig. 3). The SEM images reveal that the spent CAM has a spherical particle morphology, but its surface displays a rough texture and the presence of an amorphous layer. The less-defined granules suggest a loss of crystallinity and surface deterioration, likely due to the continuous cycling of the sample. The EDS image demonstrated a distribution of Ni (green), Mn (purple), and Co (blue) elements across the particle, with a notable predominance of Ni.

The XRD pattern of the spent CAM and the simulated pattern of α-NaFeO_2_ crystal structure are presented in Figure S3 (A). The main diffraction peaks of spent CAM match very well the characteristic peaks of a layered structure with a Rhombohedral unit cell. Extra peaks that do not correspond to the layered structure are marked as (?) and (*). The ones indicated as (*) match the expected 2theta position for a Li deficient spinel-like phase (Li_x_MO_2+y_), which has been identified as one of the main products of NMC long cycling that leads to capacity degradation. The two other peaks were not identified (?). Peak indexing for spent CAM results in a Rhombohedral unit cell with parameters a = 2.85 Å, c = 14.25 Å, and V = 100.53 Å^3^. The spent CAM did not deliver electrochemical capacity when tested in a coin cell, Figure S3(B).

### Influence of the variables studied in the hydrothermal re-lithiation process

In the hydrothermal re-lithiation process, a specific concentration of cathode powder was added to a high-pressure reactor containing LiOH at concentrations of 0.5, 2.0 and 4.0 M. The reactor was then heated to temperatures of 160 and 220 °C. The temperature and pressure were maintained throughout the reaction duration (1 and 2 h). After the reaction time elapsed, the reactor contents were filtered to separate the solid CAM from the liquid phase. The washed solid CAM was first dried in an oven and then annealed in a muffle furnace to enhance crystallinity and ensure thorough re-lithiation. After hydrothermal re-lithiation, the lithium composition of the regenerated cathode materials was measured using ICP-MS (Figure S4).

Through the analysis of variance (ANOVA) of the results, significant main effects and interaction effects were identified for concentration and temperature, while reaction time alone did not significantly affect composition of cathode material, however the influence of time on the composition of cathode material becomes relevant for specific levels of concentration, therefore its interaction with concentration is important. Regarding to the main effects about concentration, a significant main effect (*p* = 0.002) was observed indicating that different levels of lithium solution concentration led to significant variations in the composition of the cathode material. A significant main effect (*p* = 0.006) was also detected for temperature, being a critical variable that influences the composition of cathode material. However, no significant main effect (*p* = 0.172) was found for reaction time, implying its limited independent influence on the composition of cathode material.

On the other hand, the concentration-temperature interaction has a significant interaction effect (*p* = 0.005), indicating that the impact of concentration on the composition of cathode material depends on the level of temperature. Furthermore, the concentration-time interaction has also a significant interaction effect (*p* = 0.03), indicating that the impact of concentration on the composition of cathode material depends on the level of time (Figure S5). Therefore, the influence of time on the composition of cathode material becomes relevant when paired with specific levels of concentration.

Taking these results into account, the tests with the highest lithium composition, M1, M2, and M8, were selected for characterization using XRD, SEM-EDS, and electrochemical testing together with the commercial NMC 622 cathode material.

### Regenerated CAM characterization

**Fig. 4:**
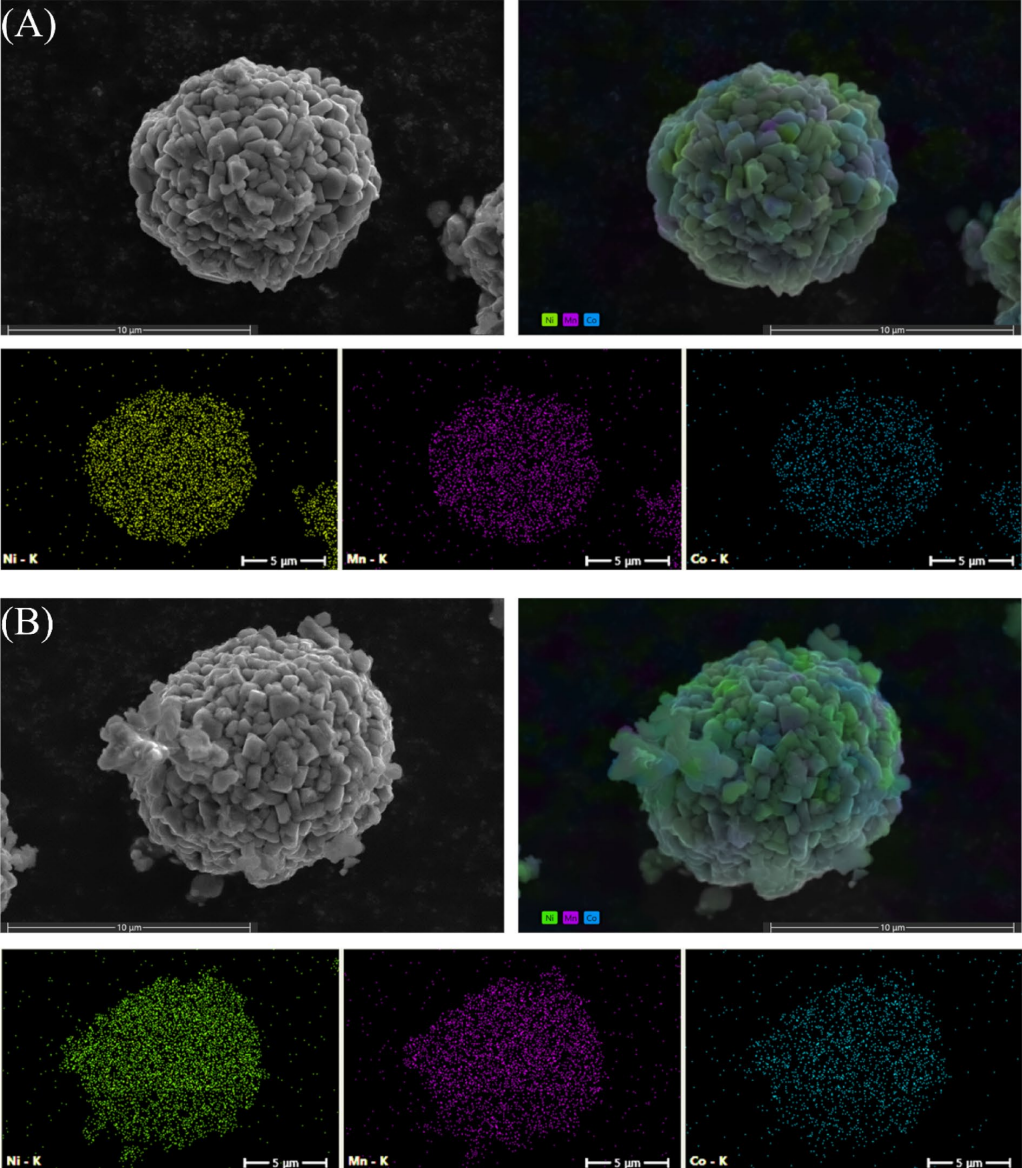

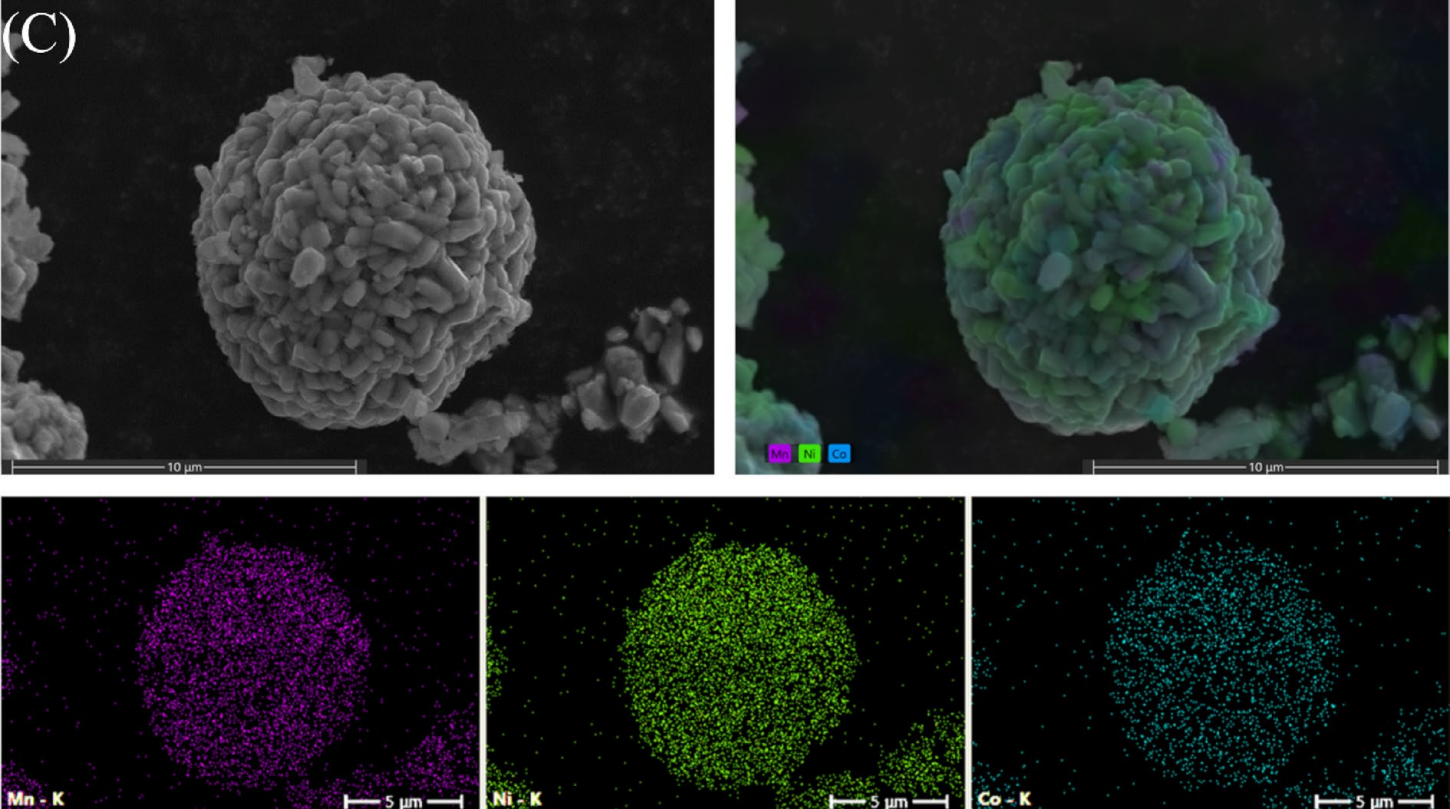
SEM images of regenerated CAM: M1 **(A)**, M2 **(B)** and M8 **(C)** and their corresponding SEM-EDS elemental map.

SEM analysis shows that the particles maintained a spherical morphology. M1 and M2 particles (Fig. 4, A and B) exhibit well-defined edges, with individual crystals displaying a more pronounced and uniform prismatic structure compared with the spent CAM in Fig. 3. M8 shows less defined particle edges with more elongated crystals (Fig. 4(C)). EDS elemental maps reveal the distribution of all relevant elements, in this case, a uniform distribution of Ni (green), Mn (purple), and Co (blue) elements across the particles for all samples, which is consistent with the material being an NMC cathode.

**Fig. 5:**
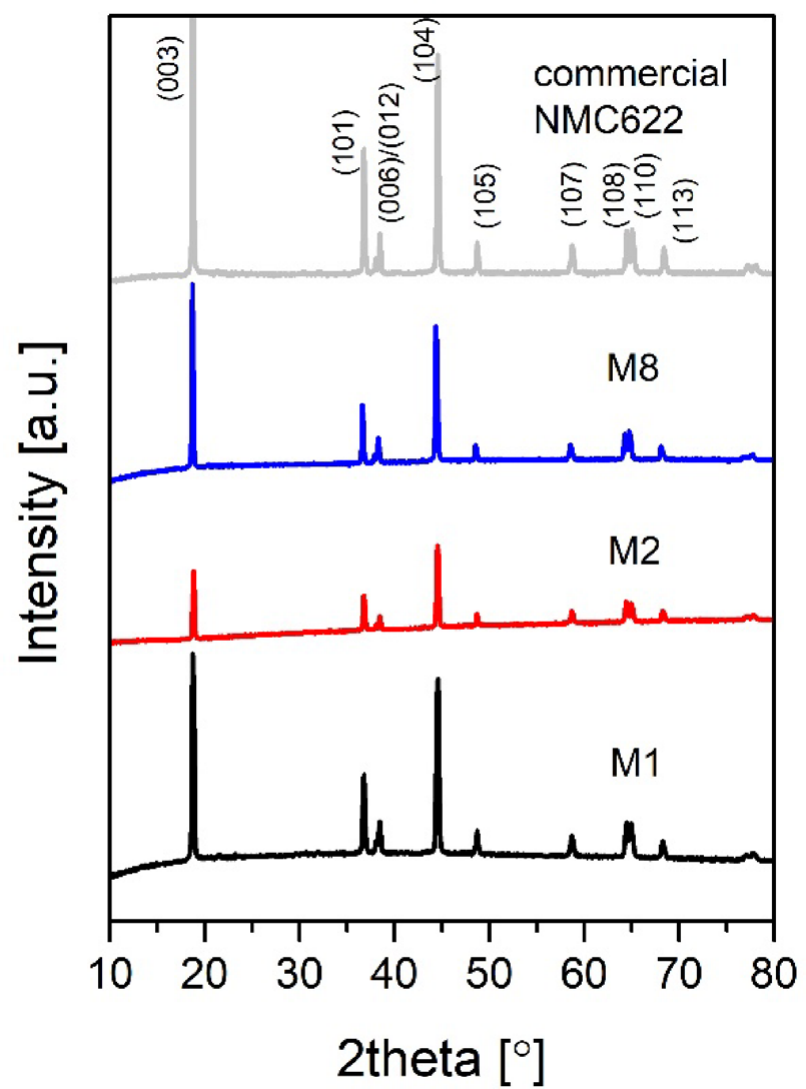
XRD patterns of regenerated and commercial CAM: M1 (black trace), M2 (red trace), M8 (blue trace), and commercial NMC622 (grey trace). Reflections characteristic of layered NMC (e.g., (003), (101), (006)/(102), (104), (108)/(110)) were used for phase identification and indexing of the R-3 m structure.

The XRD patterns of regenerated CAM (M1, M2 and M8) and commercial NMC622 are presented in Fig. 5. The diffraction patterns of regenerated CAM show the characteristic reflections of a layered R-3 m structure with no extra peaks, indicating phase-pure materials by XRD. Importantly, the hydrothermal re-lithiation plus annealing removed the impurity reflections observed in the spent CAM (Figure S3). The peak maxima for regenerated samples were considered for indexing and the resulting lattice parameters are summarized in Table 2. Along with the calculated lattice parameters (a, c, and unit cell volume), the I003/I104 ratio was evaluated as a descriptor of cation mixing between Li^+^ and Ni^2+^ in the lithium layer^22^.

As shown in Table 2, the lattice parameters and unit cell volumes for M1 and M2 are close to those of the commercial NMC622 reference. M1 and M2 were regenerated using the same LiOH concentration (4 M) and temperature (160 °C), but different hydrothermal reaction times (1 h for M1 and 2 h for M2). Sample M8 shows a slightly larger unit cell volume (101.83 Å^3^), consistent with its lower lithium content measured by ICP-MS (Figure S4) and the reported expansion of the layered lattice under lithium deficiency^23^. The I003/I104 ratios derived from the XRD patterns (Table 2) indicate that M2 exhibits more pronounced cation mixing than M1 and M8. A plausible explanation is that prolonged exposure under strongly alkaline hydrothermal conditions can promote partial surface reconstruction and transition-metal redistribution, increasing the probability of Ni^2+^ occupying Li sites after subsequent high-temperature annealing.

**Table 2:** Indexing results using a Rhombohedral unit cell for regenerated, spent and commercial samples.

Sample	M1	M2	M8	Spent CAM	NMC
a [Å]	2.86	2.86	2.875	2.85	2.86
c [Å]	14.19	14.16	14.22	14.25	14.19
Cell Volume [Å^3^]	100.80	100.81	101.83	100.53	100.67
I_003_/I_104_	1.18	0.82	1.29	2.05	2.09

### Electrochemical performance

**Fig. 6:**
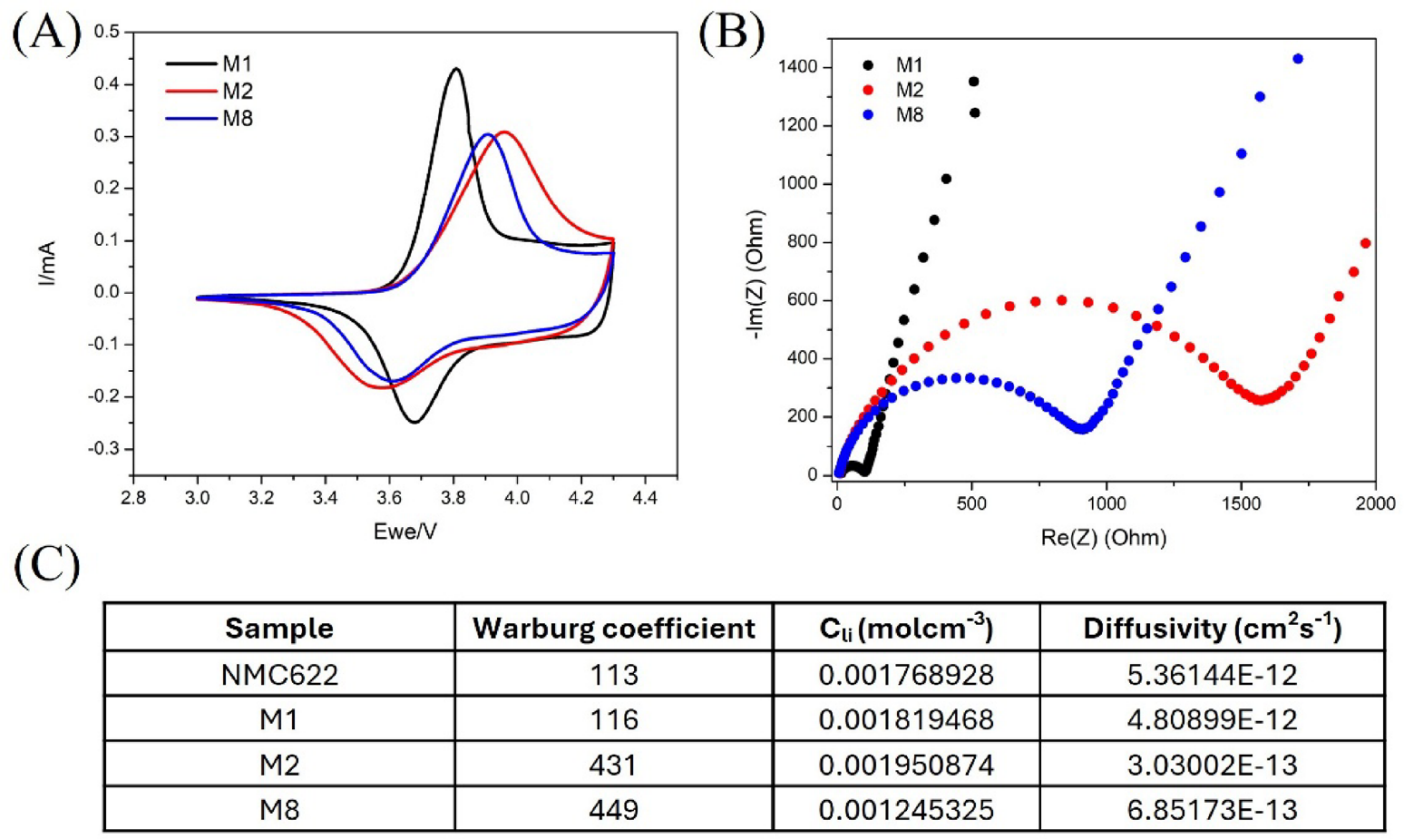
**(A)** Cyclic voltammetry and **(B)** Nyquist plots of regenerated NMC622 electrodes measured after formation cycles at 0.05 C, and (**C**) Calculated diffusivities for the regenerated samples using the Warburg coefficient obtained from Nyquist plots at low frequencies.

Cyclic voltammetry (CV) plots for the three regenerated cathodes are shown in Fig. 6(A). The three samples show a similar overall current response, but peak positions and intensities differ. The redox peaks in the anodic and cathodic regions are mainly associated with the Ni^2+^/Ni^3+^ (and Ni^3+^/Ni^4+^) redox couples, characteristic of the lithium intercalation/deintercalation process in NMC-type electrodes. M1 exhibits higher peak currents and smaller peak separation than M2 and M8, indicating faster kinetics and higher reversibility. Electrochemical impedance spectroscopy (EIS) Nyquist plots for discharged cells after activation are shown in Fig. 6(B). The spectra show the typical response of layered oxide cathodes, including a high-frequency intercept (ohmic resistance), a mid-frequency semicircle associated with interfacial/charge-transfer processes, and a low-frequency tail related to diffusion. Based on the semicircle diameter, the apparent charge-transfer resistance follows M1 < M8 < M2 (with M1 approximately 100 Ω), consistent with the CV trends. The Li diffusion coefficient for the regenerated samples was calculated using the Warburg coefficient obtained from low-frequency impedance data (Figure S5 and related equations), and the results are shown in Fig. 6(C). M1 demonstrates an ionic diffusivity in the same order of magnitude as the commercial NMC622 sample (~ 10^− 12^ cm^2^s^− 1^). Samples M2 and M8 exhibit one order of magnitude lower lithium diffusivity (~ 10^− 13^ cm^2^s^− 1^) than the M1 sample.

**Fig. 7:**
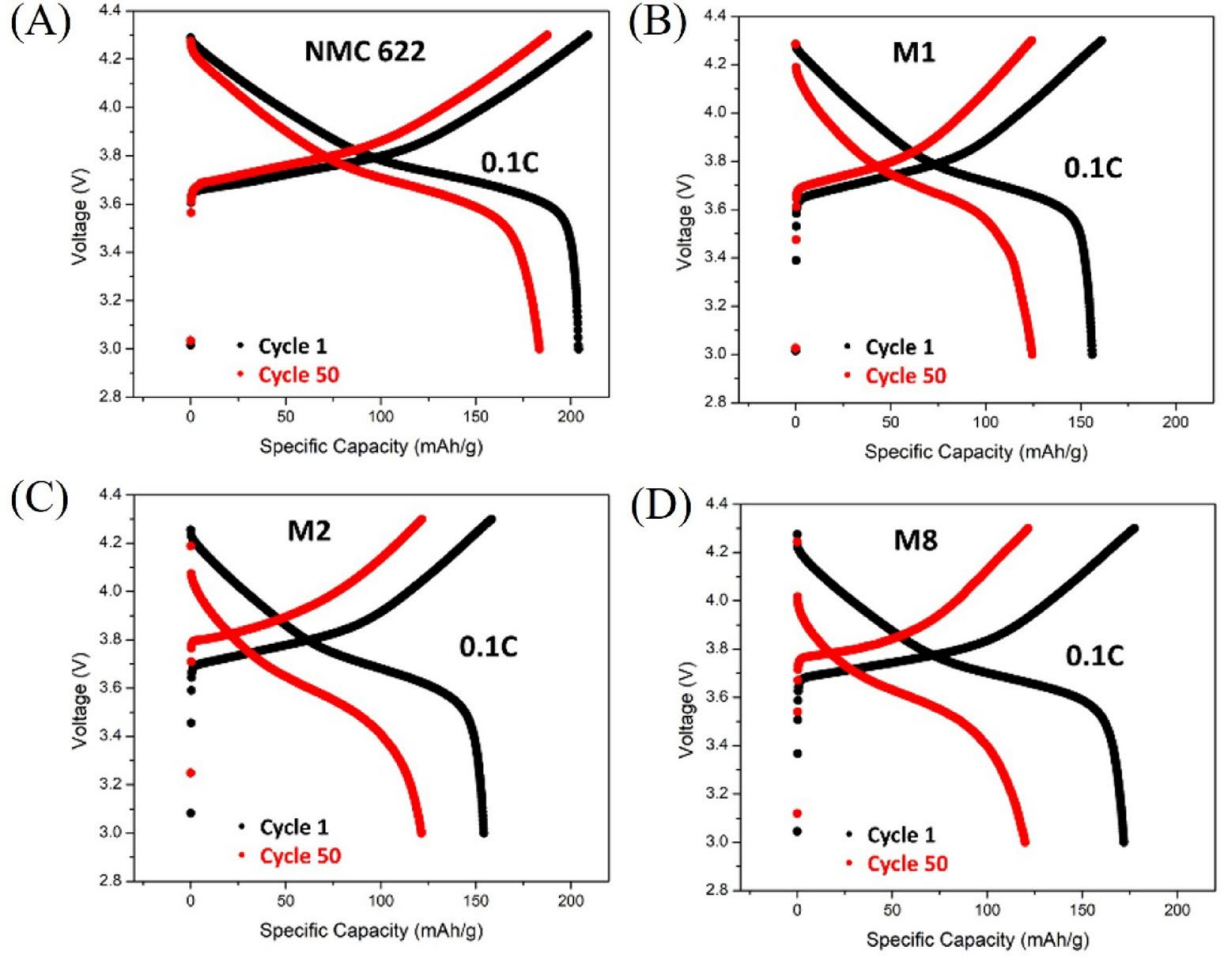
Potential vs. specific capacity of **(A)** commercial NMC622, **(B)** regenerated cathode M1, **(C)** regenerated cathode M2, **(D)** regenerated cathode M8.

Figure 7 shows voltage profiles (potential vs. specific capacity) at 0.1 C for the commercial NMC622 and the regenerated samples. All samples exhibit the characteristic charge/discharge features of layered NMC cathodes; however, the regenerated electrodes show a larger voltage gap between the charge and discharge plateaus than the commercial reference, particularly for M2, indicating increased polarization. This behaviour is consistent with the higher impedance observed by EIS (Fig. 6(B)) and the lower CV reversibility (Fig. 6(A)) for M2 and M8 relative to M1. Among the regenerated samples, M8 exhibits the highest initial discharge capacity (172 mAh/g at cycle 1) but decreases to 119.8 mAh/g after 50 cycles, whereas M1 and M2 deliver 156 mAh/g and 154 mAh/g at cycle 1, respectively. Additional diagnostics, such as differential capacity analysis (dQ/dV) and low-frequency EIS analysis (Figure S7), demonstrate that polarization increases with cycling, primarily due to a decrease in ionic diffusivity. The Nyquist plots at low frequency show a transition from a 45° slope or semi-infinite Warburg element (cycle 1) to a resistive behaviour resulting in a semicircle intercepting the real Z’ axis (cycle 150). The charge-transfer resistance shows little or no dependence on cycle number, as in the case of M8.

**Fig. 8:**
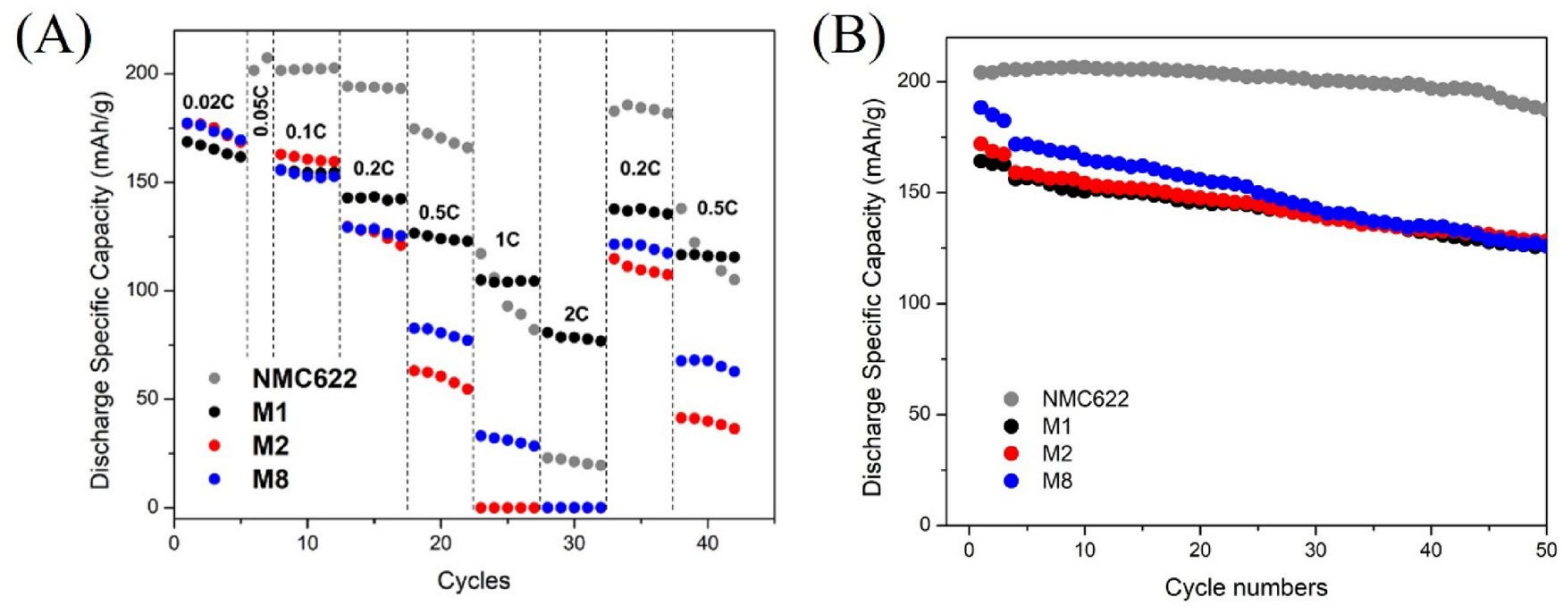
**(A)** Rate capability of M1, M2, M8 vs. commercial NMC622 in voltage window 3.0–4.3 V; **(B)** cycling stability of M1, M2, M8 vs. commercial NMC622 at 0.1 C measured after cell formation.

The rate capabilities of regenerated electrodes were investigated and compared with commercial NMC622 (Fig. 8(A)). The electrodes were charged and discharged successively at 0.02 C, 0.1 C, 0.2 C, 0.5 C, 1 C, and 2 C every 5 cycles, respectively, and tested again at 0.2 C and 0.5 C at the end of the cycling. Among the three regenerated samples, M1 exhibits higher specific capacity than the other ones, especially at 0.5 C, 1 C, and 2 C with 124 mAh/g, 104 mAh/g, and 78 mAh/g, respectively. M1 shows the highest specific capacity of 75 mAh/g at 2 C, which is even higher than the discharge capacity of the commercial sample. The cycling tests at 0.1 C for the three samples are presented in Fig. 8(B). M1, M2, and M8 show similar cycling behaviour with retention capacity of 80% for M1 and M2, and 70% for M8 after 50 cycles. Long cycling presented in Figure S8 demonstrates that the degradation behaviour of recycled samples is comparable to that of commercial NMC622.

## Discussion

### Electrochemical recovery and structural repair of re-lithiated NMC622

The results obtained for sample M1 (160 °C, 4 M LiOH, and 1 h hydrothermal reaction time, followed by annealing at 850 °C for 4 h) support the feasibility of directly regenerating Ni-rich NMC622 by restoring both lithium inventory and layered crystallinity. Structurally, M1 exhibits clear layered-type peak splitting in XRD and an I003/I104 ratio of 1.18 (Table 2), approaching the commercial reference. Electrochemically, M1 shows the lowest apparent charge-transfer resistance, high ionic conductivity, and the highest rate capability among the regenerated samples (Figs. 6 and 8). In contrast, M2 (same concentration and temperature, but longer hydrothermal time, 2 h) shows a much lower I003/I104 ratio (0.82) and higher electronic and ionic impedances, resulting in a higher polarization (Fig. 7), indicating that extended hydrothermal exposure can adversely affect cation ordering and kinetics.

A key outcome of this study is a process-structure-performance relationship across the three selected regenerated powders. The factorial design indicates that LiOH concentration and temperature have significant main effects on lithium reincorporation, while the influence of reaction time becomes relevant through interactions (Sect. 3.2). The comparison between M1 and M2 highlights that, at fixed LiOH concentration (4 M) and temperature (160 °C), increasing the hydrothermal time from 1 h to 2 h correlates with a marked increase in cation mixing (lower I003/I104) and poorer electrochemical kinetics. A plausible mechanism is that prolonged treatment in strongly alkaline media promotes surface reconstruction and transition-metal redistribution, increasing the probability of Ni^2+^ occupying Li sites after high-temperature annealing.

The findings from this work also highlight the broader sustainability advantages of direct cathode recycling (hydrothermal re-lithiation) compared to traditional hydrometallurgical or pyrometallurgical routes^24^. Recent analyses show that regenerating cathodes via a low-temperature hydrothermal process requires only a small fraction of the energy consumed by conventional recycling. For instance, Xu et al.^14^, report that a hydrothermal re-lithiation method can consume as little as ~ 4–5 MJ per kg of cathode, which is on the order of 15% of the energy demand of hydrometallurgical processing (≈ 30 MJ/kg). Pyrometallurgy is similarly energy intensive – roughly half the energy of hydrometallurgy, but still several-fold higher than direct recycling^25^. In this study, the mild reaction conditions (sub-250 °C aqueous process and moderate annealing) exemplify this low energy footprint, especially when compared to the > 1000 °C smelting and multi-step chemical extraction involved in pyro/hydrometallurgical recycling. Importantly, the comparison with hydrometallurgical and pyrometallurgical routes in this work is based on literature-reported process metrics and life-cycle assessments, rather than parallel recycling experiments using the same feedstock.

### Energy efficiency of hydrothermal direct recycling

In terms of environmental impact, direct cathode regeneration offers dramatic reductions in greenhouse gas emissions and chemical waste. A recent life-cycle assessment of Ni-rich NMC recycling found that the hydrothermal re-lithiation route produces only about 0.6 kg of CO₂ per kg of cathode recycled, whereas hydrometallurgy and pyrometallurgy emit approximately 2.3 kg and 2.16 kg CO₂/kg, respectively^26^. In other words, direct recycling of NMC622 can cut carbon emissions by roughly 74% relative to the incumbent hydrometallurgical process. The elimination of strong acids, organic solvents, and high-temperature furnaces in our process not only curtails CO₂ output, but also avoids hazardous effluents (such as metal-laden waste water or furnace slag) that require further treatment^24^^,^^26^. Moreover, direct regeneration maximizes resource recovery efficiency: nearly the entire cathode mass is directly reused as active material, rather than being reduced to constituent elements. This contrasts with pyrometallurgy, where lithium is often lost to the slag and only Ni/Co are recovered, or hydrometallurgy which must precipitate new cathode precursors from solution^27^. By preserving the embedded energy and structure of the cathode, the direct method realizes a far more circular process. Indeed, economic evaluations have shown that reconditioning cathodes in this way yields greater net benefit – one study noted that hydrothermal direct recycling of NMC was ~ 1.3 times more profitable than hydrometallurgy, partly because the output is a ready-to-use cathode material of high value (whereas traditional methods produce lower-value intermediates that need refining)^25^. This economic edge is consistent with our observations: the regenerated NMC622 from sample M1 can be directly reassembled into new cells without the costly steps of synthesizing fresh cathode powder from raw oxides. Table 3 summarise the advantages of direct recycling approach with state-of-the-art recycling technologies.

**Table 3:** Comparative Advantages of Direct Recycling vs. SoA Battery Recycling Technologies^28^.

Indicators	Direct Recycling (Hydrothermal Re-lithiation)	Hydro-/Pyrometallurgy
**Energy Consumption**	~ 4–5 MJ/kg (~ 10–20% of the energy use of conventional methods)	~ 18–30 MJ/kg
**Emissions and Waste**	~ 0.6 kg CO₂/kg cathode No toxic leachate or slag generated	> 2 kg CO₂/kg cathode Produces toxic leachate and slag (acids, smelting)
**Recovery Efficiency**	Full cathode (including lithium) recovered as reusable active material	Only metal values recovered (often excluding lithium); needs re-synthesis
**Economic Benefits**	Up to ~ 30% higher profit margin. Avoids costs of re-manufacturing	Lower profitability, especially pyrometallurgy due to energy costs

Taken together, these points emphasise that the hydrothermal direct recycling approach used for sample M1 is effective in restoring battery performance and aligns with the goals of energy conservation and environmental sustainability. Recent reviews concur that direct “repair” or “upcycling” of cathodes is emerging as the preferred route when feasible, since it outperforms pyrometallurgy and hydrometallurgy in energy efficiency, cost, and emissions according to contemporary assessments^29-31^. The results of this work reinforce those conclusions, providing a practical demonstration that an optimized hydrothermal re-lithiation can regenerate Ni-rich cathodes in a cleaner and more efficient manner than traditional recycling processes.

## Conclusions

An efficient direct recycling process for regenerating spent cathode materials from EoL LIBs in EVs has been developed. This process involves discharging and disassembling before separating the batteries modulus and cells manually to obtain the cathode material, then performing a hydrothermal re-lithiation reaction to reintroduce lithium into the spent cathode materials, followed by an annealing step to obtain regenerated cathode materials for battery manufacturing. Before the direct regeneration experiment, degradation issues such as lithium deficiency and rough particles were identified. The lithium stoichiometry was restored after the hydrothermal re-lithiation and annealing processes, and the particles regained their crystalline structure. Synthesis conditions defined for M1, M2 and M8 effectively removed the impurity phases from spent CAM, delivering unit cell lattice parameters and volumes aligned with the commercial NMC622 used as a standard; only M8 shows a slightly larger unit cell volume that we correlate to a small but significant Li deficiency in the crystal structure, as observed by ICP analyses. The regenerated electrodes were subjected to CV, EIS, and galvanostatic charge/discharge testing, showing the typical behaviour of NMC associated with the Ni^3+^/^2+^ redox couple. The best performing sample was M1 since it delivered the highest discharge capacities of 124 mAh/g, 104 mAh/g, and 78 mAh/g at 0.5 C, 1 C, and 2 C, respectively, and a capacity retention of 80% after 50 cycles at 0.1 C. This enhanced performance can be correlated with a more ordered crystal structure, as indicated by the intensity ratio I003/I104 (1.18 for M1), and with its higher ionic diffusivity (4.8 × 10–12 cm^2^s^− 1^), compared to M2 and M8 (~ 10^− 13^ cm^2^s^− 1^). For a given concentration (4 M) and reaction temperature (160 °C), the number of defects in the NMC crystal structure increases as a function of reaction time, from 1.18 for 1 h to 0.82 for 2 h. Overall, we propose an efficient direct recycling process based on the hydrothermal re-lithiation reaction, which should consider a lower reaction temperature of 160 °C for 1 h using a salt concentration of 4 M (in aqueous media) to ensure full NMC lithiation and minimal structural defects to maximize the electrochemical performance of the regenerated CAM.

## Supplementary Information

Below is the link to the electronic supplementary material.

Supplementary Material 1

## Data Availability

The datasets used and/or analysed during the current study available from the corresponding author on reasonable request.
