# Supplementary material for: Direct recycling of end-of-life lithium-ion batteries cathode active materials by hydrothermal route
Source: Sci Rep. 2026 Mar 2;16:11594. doi: 10.1038/s41598-026-41973-7 (PMC13057244; doi:10.1038/s41598-026-41973-7)
Supplement: Supplementary file 1 — Supplementary Material 1 [file 41598_2026_41973_MOESM1_ESM.docx]

# Supplementary Material


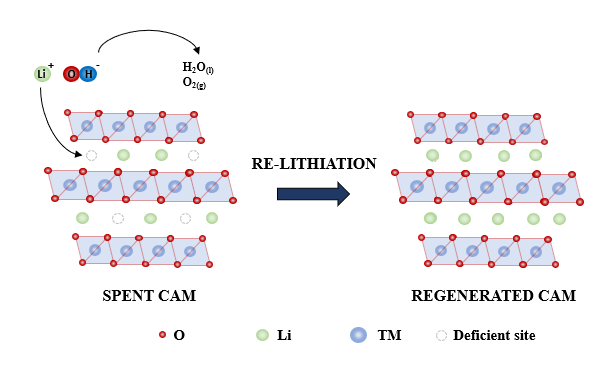


**Figure S1.** Illustration of the re-lithiation reaction mechanism.


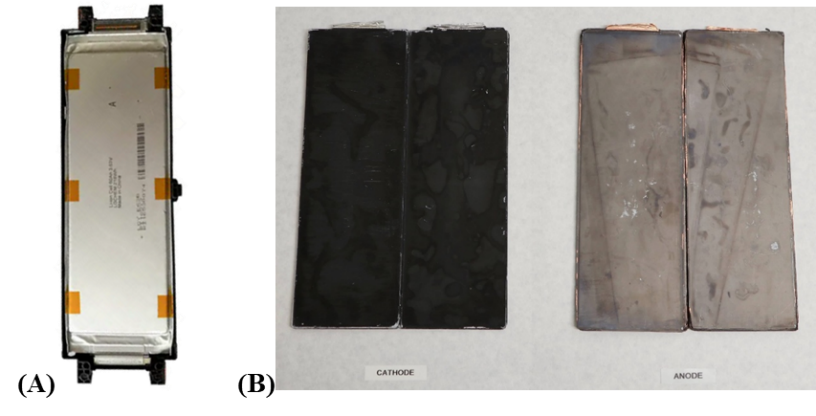


**Figure S2.** **(A)** LG Chem E63B cell; **(B)** Cathode and anode foils after manual disassembling. Source: Photo by the author.

**Table S1.** Experimental conditions for the hydrothermal tests.

| **Test** | **LiOH Concentration (M)** | **Temperature (°C)** | **Time (h)** |
| --- | --- | --- | --- |
| **1** | 4.0 | 160 | 2 |
| **2** | 4.0 | 160 | 1 |
| **3** | 4.0 | 220 | 2 |
| **4** | 4.0 | 220 | 1 |
| **5** | 2.0 | 160 | 2 |
| **6** | 2.0 | 160 | 1 |
| **7** | 2.0 | 220 | 2 |
| **8** | 2.0 | 220 | 1 |
| **9** | 0.5 | 160 | 2 |
| **10** | 0.5 | 160 | 1 |
| **11** | 0.5 | 220 | 2 |
| **12** | 0.5 | 220 | 1 |


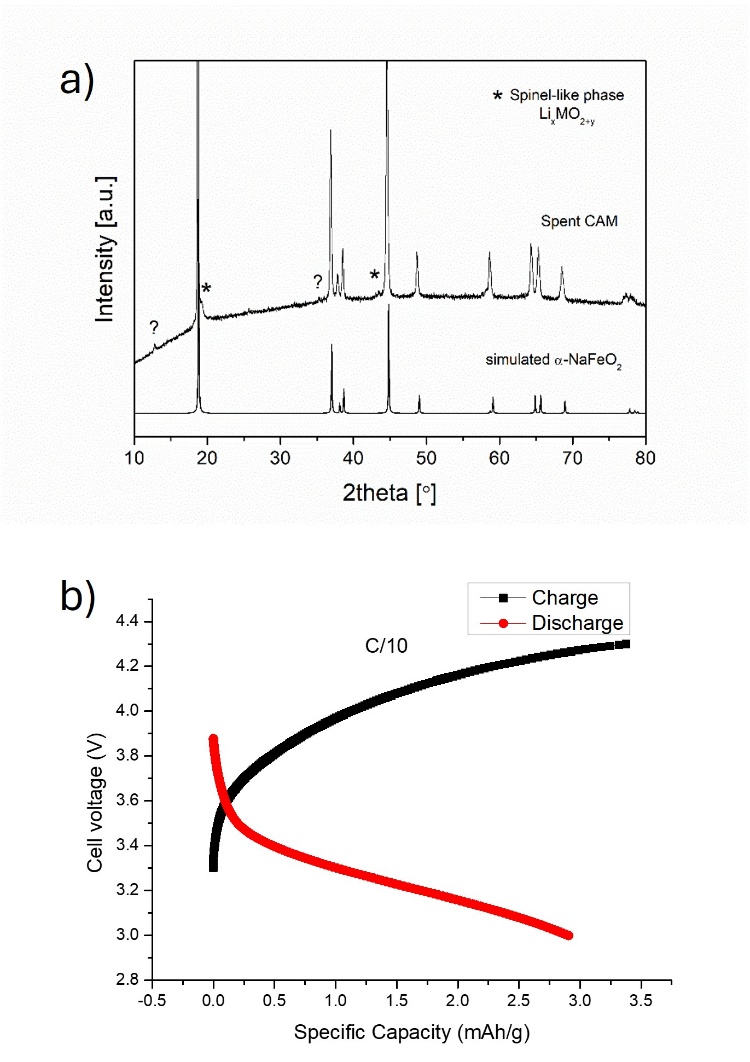


**Figure S3 (A)** XRD results of the spent CAM and (simulated) diffraction pattern of the α-NaFeO_2_ structure; (B) voltage vs specific capacity for the spent CAM (as received).


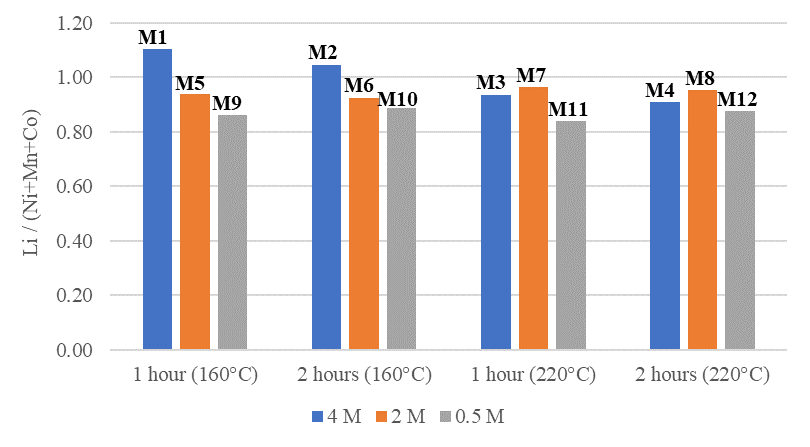


**Figure S4.** Composition of the regenerated cathode material.


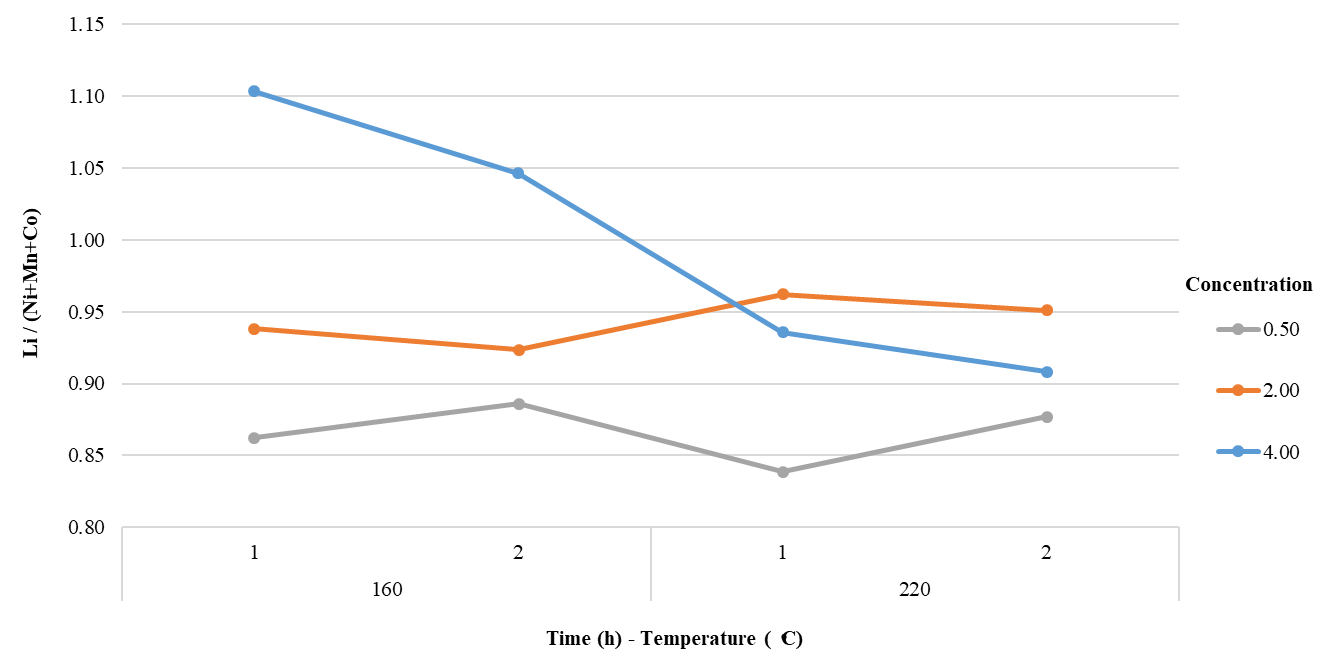


**Figure S5**. Interaction effect between concentration, temperature, and time.

**Li diffusivity calculation using EIS data:**

The Nyquist plot for regenerated samples shows the typical response of layered oxide cathodes, including a high-frequency intercept (ohmic resistance), a mid-frequency semicircle associated with interfacial/charge-transfer processes, and a low-frequency tail related to diffusion, which is associated with a Warburg element. Figure S5 a) shows the Nyquist plot for M1 electrodes and the equivalent impedance equivalent circuit used to fit the data, and b) typical Z’ vs ω^-0.5^ used to calculate the Warburg coefficient (σ).


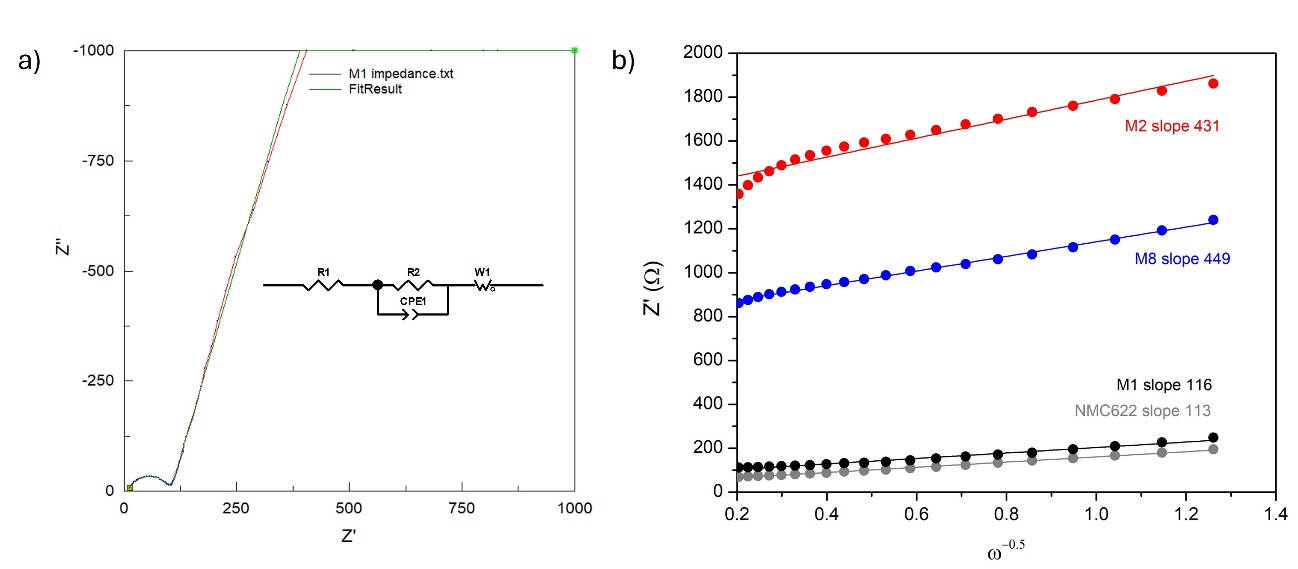


**Figure S6** Fit to Nyquist plot for M1 sample and b) Warburg coefficient calculation

First, a linear regression analysis was performed between the real part of the impedance (Z’) and the reciprocal of the square root of the angular frequency (ω^-0.5^) in the low frequency region (Figure S6 b). The Warburg coefficient is obtained from the slope of this fitted line, where ω represents the angular frequency in the low-frequency domain, Equation 1. The Li diffusion coefficient (D_Li_) is directly related to the Warburg coefficient (σ) and can be calculated using Equation 2[1]:

$Z_{r}=\sigma\times\omega^{-0.5}+R_{ct}$ Equation 1

$D_{Li}=\frac{R^{2}\times T^{2}}{2\times A^{2}\times n^{4}\times F^{4}\times C_{Li}^{2}\times\sigma^{2}}$ Equation 2

Where R is the universal gas constant, T is the absolute temperature, A is the active surface area of the electrode, n is the number of electrons involved in the redox reaction per molecule, F is the Faraday constant, C_Li_ is the molar concentration of Li intercalated in the regenerated NMC622 electrodes, and σ is the Warburg coefficient.


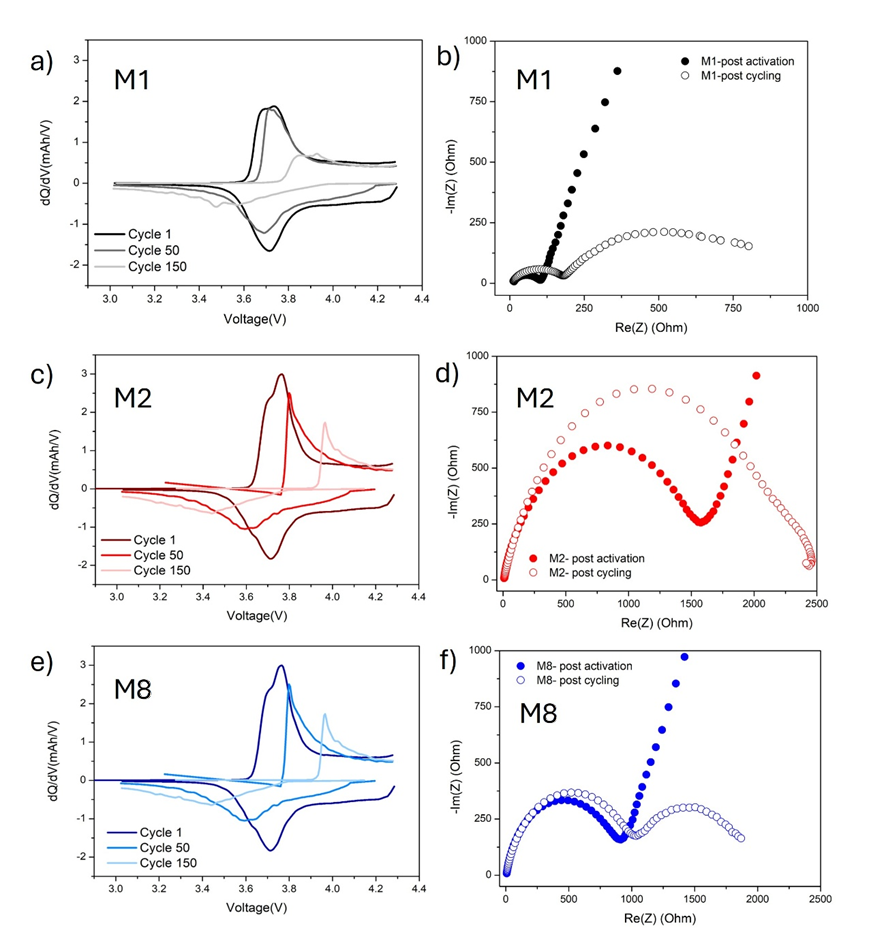


**Figure S7**. dQ/dV and EIS plots of M1, M2, and M8 electrodes measured as a function of coin-cell cycling. Post-activation corresponds to cycle 1 and post-cycling to cycle 150. EIS plots were measured in discharged samples.


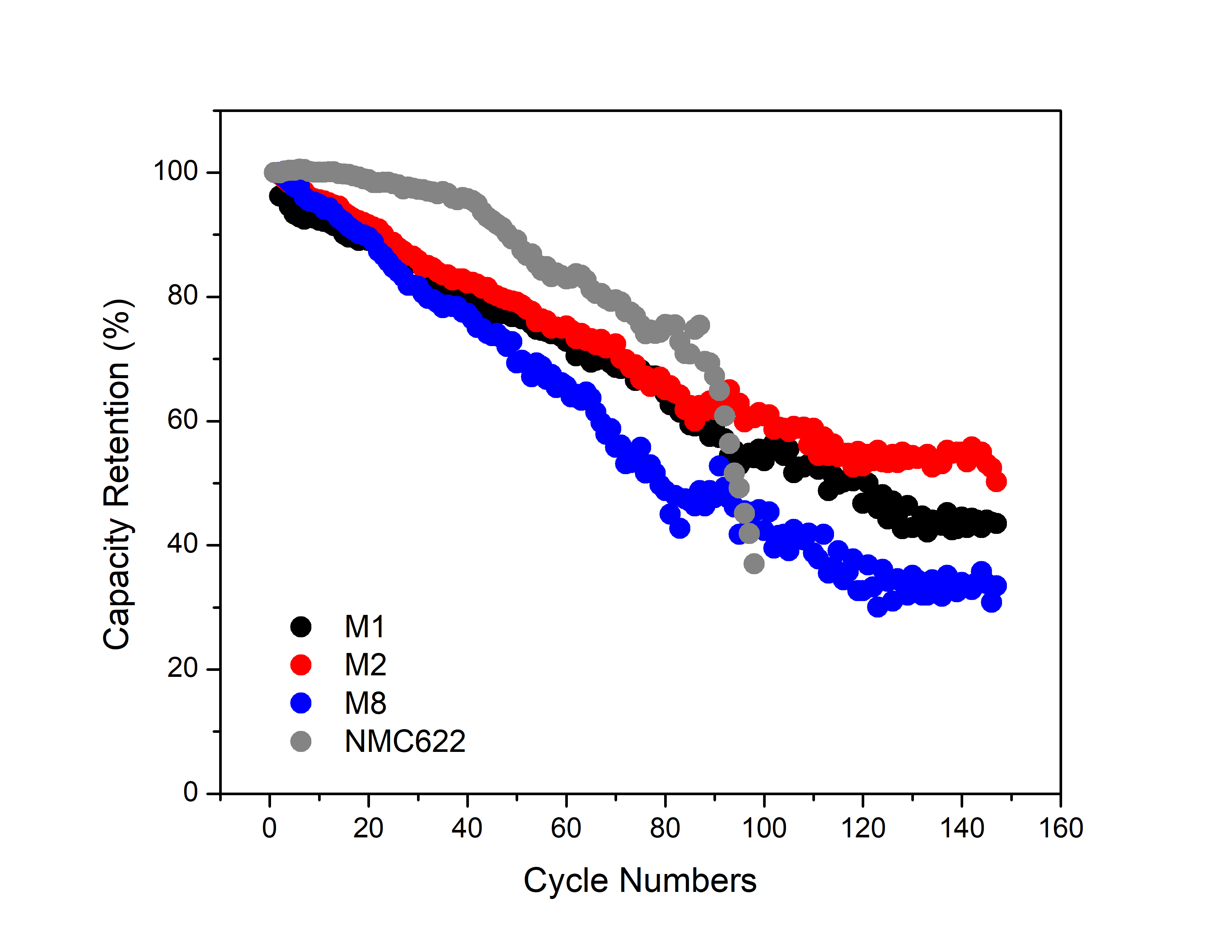


**Figure S8**. Capacity retention (%) vs cycle number for NMC622, M1, M2, and M8 electrodes.

[1] I. Stenina, “Composite cathodes based on Lithium-ion phosphate and N-doped carbon materials”, Batteries, 8(12), 256 (2022), DOI:[10.3390/batteries8120256](https://doi.org/10.3390/batteries8120256)
